# Two hundreds cases of ASIA syndrome following silicone implants: a comparative study of 30 years and a review of current literature

**DOI:** 10.1007/s12026-016-8821-y

**Published:** 2016-07-13

**Authors:** Maartje J. L. Colaris, Mintsje de Boer, Rene R. van der Hulst, Jan Willem Cohen Tervaert

**Affiliations:** 1 Faculty of Health, Medicine and Life Sciences, Maastricht University, Maastricht, The Netherlands; 2 Reconstructive, Plastic and Hand Surgery, Maastricht University Medical Center, Maastricht, The Netherlands; 3 Clinical and Experimental Immunology, Reinaert Clinic, Maastricht, The Netherlands

**Keywords:** Breast implants, Silicones, Autoimmunity, Autoinflammatory, Fibromyalgia, ASIA, Chronic fatigue syndrome (CFS)

## Abstract

In this study, we compared one hundred patients with autoimmune/inflammatory syndrome induced by adjuvants (ASIA) due to silicone implant incompatibility syndrome diagnosed in 2014 in Maastricht, the Netherlands, with one hundred historical patients with adjuvant breast disease diagnosed in the Baylor College of Medicine, Houston, USA, between 1985 and 1992. Similarities and differences between these two cohorts were identified to determine whether the spectrum of silicone-related disease changed during the last 30 years. Patients with complaints possibly due to silicone-filled breast implants were prospectively examined in the Reinaert Clinic, Maastricht, the Netherlands between January 2014 and October 2014. All patients were evaluated for the fulfilment of ASIA criteria. Results were compared to results of the Baylor College cohort and 18 other reviewed historical cohorts. Clinical manifestations between the Maastricht and Baylor College cohorts were comparable. Fatigue was observed in 98 current patients and in 95 historical patients. Arthralgia was observed in 91 versus 81 historical patients. Myalgia was observed in 54 versus 91 patients. Cognitive impairment was observed in 78 versus 81 patients, pyrexia was observed in 64 versus 52 patients, sicca complaints in 73 versus 72 patients and severe neurological manifestations in 20 versus 32 patients. From the 54 patients who underwent removal of their silicone breast implant, 50 % (*n* = 27) of the patients experienced improvement of complaints after explantation of the implant. Also, in the 18 reviewed historical cohorts, similar clinical manifestations were described. Our findings suggest that no major changes were present in the observed clinical manifestations between the Maastricht and Baylor College cohorts. Also, despite changes in the principal constituents of the silicone implants during the past fifty years, silicone remained an adjuvant that may ‘bleed’ and subsequently may be a chronic stimulus to the immune system resulting in similar clinical manifestations as observed in the Maastricht cohort, the Baylor College cohort and 18 other large cohorts of patients. We therefore conclude that silicone-related disease has not changed during the last 30 years.

## Introduction

The safety of silicone-containing breast implants has been challenged since their introduction [1, 2], even though the principal constituents of implants have changed during the last fifty years. Well-known local complications of silicone-filled implants are capsular contracture, allergic reaction and autoimmune diseases [3–8]. In addition, there is evidence for an increased occurrence of a rare form of non-Hodgkin lymphoma, i.e. anaplastic large T cell lymphoma [9–11]. Furthermore, we recently described the increased occurrence of a deficient humoral immune system in patients exposed to silicone-filled breast implants [3]. Interestingly, it is at present still controversial whether silicone-filled breast implants increase the risk of autoimmunity [3, 12, 13]. Nearly five decades after the first description of a syndrome called ‘adjuvant breast disease’ [1, 2] it was recognized that patients develop a specific disease that cannot be classified as a classic connective tissue disease (CTD) and it was proposed to label these patients as suffering from ‘autoimmune/inflammatory syndrome induced by adjuvants’ (ASIA) due to ‘Silicone Implant Incompatibility Syndrome’ (SIIS) [3, 14]. Whether ASIA due to SIIS [3, 15] is actually the same disease as the previously described adjuvant breast disease is at present still uncertain [2, 16]. To study this, we compared 100 consecutively diagnosed patients with ASIA due to SIIS with 100 historical patients described as suffering from ‘adjuvant breast disease’.

## Materials and methods

Two groups of patients with complaints due to silicone-containing breast implants are compared. A cohort of one hundred patients analysed for silicone breast implant-related complaints in 2014 in the Netherlands (‘Maastricht cohort’) and a cohort of one hundred patients diagnosed at Baylor College, Houston, Texas, USA, with ‘Adjuvant Breast disease’ due to silicone breast implants or silicone fluid injections between 1985 and 1992 as described in 1994 by Shoaib et al. (‘Baylor College cohort’) [16].

Matching criteria between the two cohorts are Shoenfeld’s criteria for the diagnosis of ASIA (Table 1). Patients who developed complaints after receiving silicone breast implants were referred to and evaluated by JWCT between January 2014 and October 2014 for prospective analysis (‘Maastricht cohort’). All patients received a careful evaluation of their complaints, their medical history and a physical examination [3, 17]. A diagnosis of ASIA was made when Shoenfeld’s criteria for this syndrome were fulfilled [14]. Explicit four major and four minor criteria were evaluated; the patient was considered having ASIA when either two major or one major and two minor criteria were present (Table 1). The first consecutive hundred patients who fulfilled Shoenfeld’s criteria for the diagnosis of ASIA were included in this study. Patients underwent laboratory measurements of immunoglobulins, antinuclear antibodies (ANA) and IgM rheumatoid factor (RF) [3, 18]. In addition, extractable nuclear antigen (ENA), antineutrophil cytoplasmic antibodies (ANCA), anticyclic citrullinated peptide (anti-CCP) were measured and several other autoantibodies (e.g. anticardiolipin antibodies) were measured in patients clinically suspected of suffering from specific autoimmune diseases [3]. Immunoglobulins IgG, IgA and IgM were tested by latex-enhanced homogenous immunoassay by antigen–antibody complex with spectrophotometry by Turbidimetry, Roche Cobas, Roche, Basel, Switzerland. Antinuclear antibodies were tested by indirect immunofluorescence on HEp-2000 cells (Immuno Concepts, Sacramento, CA) [17]. Serum samples were screened in a dilution of 1:80 [19]. IgM rheumatoid factor was tested by FEIA (Phadia ImmunoCAP 250, ThermoFisher Scientific, Freiburg, Germany) [20]. If explantation of silicone implants was performed, patients were reassessed by JWCT to document whether changes of complaints had occurred after explantation. Also, this assessment focussed on the different clinical manifestations of Shoenfeld’s ASIA criteria. The comparative group of patients, described by Shoaib et al. [16] in 1994 was composed of 100 symptomatic women with human adjuvant breast disease due to silicone incompatibility. These patients were evaluated for their symptoms primarily by an outside plastic surgeon and secondly by a neurologist in the Baylor College of Medicine (‘Baylor College Cohort’).

**Table 1:** Criteria for the diagnosis of ASIA

*Major criteria*
• Exposure to an external stimulus (infection, vaccine, silicone, adjuvant) prior to clinical manifestations
• The appearance of ‘typical’ clinical manifestations:
- Myalgia, Myositis or muscle weakness
- Arthralgia and/or arthritis
- Chronic fatigue, un-refreshing sleep or sleep disturbances
- Neurological manifestations (especially associated with demyelination)
- Cognitive impairment, memory loss
- Pyrexia, dry mouth
• Removal of inciting agent induces improvement
• Typical biopsy of involved organs
*Minor criteria*
• The appearance of autoantibodies or antibodies directed at the suspected adjuvant
• Other clinical manifestations (i.e. irritable bowel syndrome)
• Specific HLA (i.e. HLA DRB1, HLA DQB1)
• Evolvement of an autoimmune disease (i.e. multiple sclerosis, systemic sclerosis)

*ASIA* autoimmune/inflammatory syndrome induced by adjuvants

### Statistics

For statistical analysis of results, a two-group Chi-square test with a 0.05 two-sided significance level was used (SPSS 22.0 software, IBM Corp, Armonk, NY).

### Review

We performed a literature search in PubMed, MEDLINE, EMBASE and the Cochrane Database of Systematic Reviews in February 2016. Additional citations were solicited from references in selected articles. The searches combined the following main terms: silicone breast implant, silicone adverse effects and the terms silicone-related symptom complex, adjuvant breast disease, human adjuvant disease, ASIA syndrome were separately added to the main two search terms.

We included articles focussing on patients with breast implants who were experiencing complaints that were ascribed to their silicone breast implants. Articles focussing on well-defined diseases, such as autoimmune or connective tissue diseases, were excluded. Articles in the period from January 1960 to the present time, in English were included. For all the included studies, the clinical manifestations in patients with silicone-related complaints were collected. Only case series (minimal 30 patients) were included, and no case reports were included. Studies focussing on malignancies in the breast (diagnostics, therapy or reconstruction) and implant failure (rupture, infection, capsular formation) were excluded.

## Results

### Patient demographics

The Maastricht cohort consists of 99 female patients and 1 transgender. All patients are exposed to silicone gel-filled breast implants. The median age at time of implantation was 33 years (14–56 years), and the median age at onset of clinical symptoms was 41 years (20–68 years). The median latency period from time of implant until onset of clinical symptoms was 4 years (range 1–39 years). The median age at the time of diagnosis was 49 years (27–72 years). The median time between implant and diagnosis was 13 years (2–43). The comparative group of patients, the Baylor College cohort described in 1994, existed of 100 symptomatic women with either silicone breast implants (*n* = 97) or silicone fluid injections (*n* = 3). Median age was comparable to the median age of the patients from the Maastricht cohort (Table 2).

**Table 2:** Patient demographics of both the cohorts including reasons for implantation

Variable	1994	2014
	Years (range)	Years (range)
Median age at time of evaluation	NA	49 (27–72)
Median age at time of implantation	32 (19–52)	33 (14–56)
Median age at onset of symptoms	38 (23–57)	41 (20–68)
Median age at time of diagnoses	NA	49 (27–72)
Median latency period	6 (0–24)	4 (1–39)
Median time between implant and diagnosis	NA	13 (2–43)
Median age at time of removal	44 (30–59)	49 (31–68)

Reasons for implantation	1994	2014
	*N* (%)	*N* (%)
Cosmetic purposes	68	80
Malignant tumour	4	14
Benign tumour	28	3
BRCA positive	NA	3

*NA* not applicable

In both cohorts, patients received various sorts of implants from different manufacturers. All implants, however, were silicone gel-filled breast implants.

In both cohorts, approximately 70–80 % of the patients received silicone breast implants for cosmetic reasons. Other reasons for implantation were reconstruction after benign or malignant tumour removal, or reconstruction after preventive ablation due to a BRCA mutation (Table 2).

### Local manifestations and complications

In the Maastricht cohort, local problems were frequently observed: capsular contracture (*n* = 29), sweating and/or leakage of the silicone implant (*n* = 13), implant rupture (*n* = 25), dislocation of the implant (*n* = 3) or local tenderness (*n* = 4). Furthermore, 70 patients had painful lymphadenopathy involving the axillary regions, often with cervical and/or inguinal lymphadenopathy as well.

In the Baylor College cohort of 1994, 76 patients suffered from local problems defined as capsular contracture, tenderness, soreness or pain of the breasts, burning and swollen breasts, infections, numbness of the nipples or discharge from the nipples. Fifty-eight patients had lymphadenopathy.

### Clinical symptoms

In the Maastricht cohort, arthralgia (*n* = 91) and chronic fatigue (*n* = 98) are most often observed, followed closely by the other typical clinical manifestations of ASIA (Table 3). Other manifestations that were scored in the Maastricht cohort are Raynaud’s phenomenon, irritable bowel syndrome (IBS), recurrent respiratory tract infections, recurrent cystitis, livedo reticularis and allergies (Table 4). In the Baylor College cohort, comparable numbers of clinical manifestations have been identified (Tables 3, 4).

**Table 3:** Presence of clinical manifestations in both cohorts

Symptom	1994	2014	*P* value
	*N* (%)	*N* (%)	
Myalgia, Myositis or muscle weakness	91	54	<.001
Arthralgia and/or arthritis	81	91	.04
Chronic fatigue, un-refreshing sleep or sleep disturbances	95	98	.25
Neurological manifestations (especially associated with demyelination)	32	20	.05
Cognitive impairment, memory loss	81	78	.60
Pyrexia	52	64	.09
Dry eyes and/or dry mouth (sicca)	72	73	.87

**Table 4:** Presence of other clinical manifestations in both cohorts

Symptom	1994	2014	*P* value
	*N* (%)	*N* (%)	
Raynaud’s phenomenon	58	30	<.001
Irritable bowel syndrome	NA	17	–
Recurrent respiratory tract infections	NA	54	–
Recurrent cystitis	NA	36	–
Livedo reticularis	62	28	<.001
Allergies	52	52	NS

*NA* not applicable

### Laboratory findings

In the Maastricht cohort, fewer patients had antinuclear antibodies when compared to the cohort as described in 1994 (Table 5). In eight patients laboratory measurements for IgM rheumatoid factor could not be performed, and in two patients laboratory measurements for immunoglobulins could not be performed, due to an inadequate volume of serum. Herefore this data is reported as missing data.

**Table 5:** Laboratory findings

Measurement	1994	2014
	*N* (%)	*N* (%)
Increased immunoglobulins	13/76 (17)	14/98 (14)
Decreased immunoglobulins	24/76 (32)	13/98 (13)
ANA	33/93 (36)	5/100 (5)
RF	10/90 (11)	4/92 (4)

Immunoglobulins include total IgG, IgA and IgM, *ANA* antinuclear antibodies, *RF* IgM rheumatoid factor

### Presence of autoimmune diseases

In the Maastricht cohort, 34 patients were diagnosed with an autoimmune disease (Table 6). The presence of autoimmune diseases has not been described in the Baylor College cohort.

**Table 6:** Diagnostic findings in 100 patients with silicone-filled breast implants and ASIA in the 2014 cohort

Symptom	*N* (%)
RA	4
CTD	18
Vasculitis	5
Granulomatous disease	3
Other	7

*RA* rheumatoid arthritis, *CTD* connective tissue disease (systemic sclerosis *n* = 2, undifferentiated connective tissue disease *n* = 1, Sjogren’s syndrome *n* = 5, antiphospholipid syndrome *n* = 7, systemic lupus erythematosus *n* = 3); granulomatous disease (Crohn *n* = 1, sarcoidosis *n* = 2); Other autoimmune diseases (multiple sclerosis *n* = 1, M. Hashimoto *n* = 2, polychondritis recidivans *n* = 1, pernicious anaemia *n* = 1, lichen planus *n* = 1 and neuralgic amyotrophy *n* = 1)

### Implant removal

In the Maastricht cohort, 54 patients underwent removal of their silicone breast implant. Of these, 50 % (*n* = 27) of the patients experienced improvement of complaints after explantation of the implant. The median age at removal was 49 years (range 31–68). Symptoms such as fatigue, arthralgia, myalgia, sicca and pyrexia improved in most patients after explantation. In seven patients, however, the improvement was only observed temporarily, with a relapse of complaints after several weeks follow-up.

In the Baylor College cohort, 96 patients underwent explantation of the implant. The median age of the patients at explantation was 44 years (range 30–59). Whether these patients experienced improvement of their complaints after explantation was not described in the paper.

### Statistics

We found significant differences between the two cohorts for the following clinical manifestations: myalgia, myositis or muscle weakness (*p* < .001) and arthralgia and/or arthritis (*p* = .04). Other clinical manifestations were not found to be significantly different: chronic fatigue (*p* = .25), neurological manifestations (*p* = .05), cognitive impairment (*p* = .60), pyrexia (*p* = .09) and sicca (*p* = .87). Significant differences, however, were also observed between the two cohorts regarding livedo reticularis (*p* < .001) and the occurrence of Raynaud’s phenomenon (*p* < .001) but not in the occurrence of allergies.

## Discussion

The objective of this study was to explore whether ASIA due to SIIS is the same disease as the previously described adjuvant breast disease [16]. The results in this study show that clinical findings in patients with silicone breast implants in the current Maastricht cohort and the historical Baylor College cohort are more or less identical (Table 3). The typical clinical manifestations (major criterion 2 of the ASIA syndrome, Table 1) are clearly present in both cohorts. In the Maastricht cohort, almost all patients presented with fatigue, in combination with arthralgia and/or myalgia. These symptoms were nearly always accompanied with pyrexia, cognitive impairment and/or sicca complaints.

There are, however, differences between both cohorts. Myalgia was less frequently observed in the Maastricht cohort in comparison with the Baylor College cohort, whereas arthralgia/arthritis was more frequently observed in the Maastricht cohort. The difference could be due to the fact that arthralgia and myalgia are difficult to distinguish. Both symptoms have a musculoskeletal origin; the true origin of the pain is therefore sometimes difficult to establish. Another explanation might be that Shoaib et al. [16] (a neurologist) did a more complete neurological work-up, whereas the auto-immunologist in Maastricht performed a more complete rheumatological work-up [3]. Shoaib et al. have performed MRI’s, muscle biopsies, electromyograms and specific measurements of autoantibodies (anti-GM1) to detect neurological diseases. These factors possibly explain why myalgia/myositis/muscular weakness and neurological manifestations scored higher in the Baylor College cohort.

Comparing the frequency of positive antinuclear antibodies and IgM rheumatoid factor between the two cohorts, less autoantibodies were detected in the Maastricht cohort compared to the Baylor College cohort. It could be that current silicone breast implants are less immunogenic, explaining fewer autoantibodies in the Maastricht cohort. Otherwise, the definitions of a positive ANA and/or positive rheumatoid factor may have been different. Unfortunately, from the Baylor College cohort, the exact description of ANA and rheumatoid factor measurement and interpretation is lacking and detailed information about cut-off values for ANA and IgM-RF are not described in the paper by Shoaib [16].

In another study, in 156 women with adjuvant breast disease performed at the University of Missouri-Columbia in 1993, a positive ANA was found in 22 % of the patients and a positive IgM-RF in 9 % of patients [21]. These results resemble more closely the results of autoantibody testing in the Maastricht cohort, suggesting that antigenicity of the breast implants did not change during the last 30 years. Finally, in the Maastricht cohort, the course of disease has been evaluated after explantation of the silicone breast implant. Improvement of complaints occurred in 50 % of the patients. In another study, conducted by Peters et al. in 1997, a similar outcome was reported: 58 % of their patients had fewer symptoms after explantation, whereas 74 % of the patients declared that they ‘felt better’ and that their quality of life had improved [22].

### Review of current literature

 Based on the similarities between the Maastricht and the Baylor College cohort, we decided to conduct a critical review of the literature, to study whether these similarities were also present in patients who have presented with silicone-related complaints in other studies. The results are presented in Table 7.

**Table 7:** Summary of identified clinical manifestations in reviewed cohorts

References	Cohort (N)	Fatigue	Arthralgia	Myalgia	Sicca	Pyrexia	Neurological symptoms	Memory/cognitive impairment	Other symptoms
Maijers [15]	80	X	X	X	NR	NR	NR	X	Dermatological problems, alopecia, night sweats, depression, GI-symptoms
Cohen Tervaert [3]	32	X	X	X	NR	X	NR	NR	Asthenie
Contant [23]	57	NR	X	NR	X	NR	NR	NR	Raynaud, headache, dizziness, palpitations, diarrhoea transpiration
Contant [24]	63	NR	X	NR	X	NR	X	X	Raynaud, headache, palpitations, diarrhoea, transpiration, night sweats, rashes
Shoaib [25]	26	NR	X	X	X	X	X	NR	Headache, skin rash, Raynaud’s, hair loss, allergies, sensitivity to sunlight and lymph-adenopathy
Cuellar [26]	300	X	X	NR	X	X	X	X	Headache, night sweats, diarrhoea, recurrent infections, dyspnoea, angio-oedema and shoulder pain
Gaubitz [27]	90	X	X	X	X	NR	NR	NR	Night sweats, Raynaud, tingling, photosensitivity, headache, dyspnoea, depression, hoarseness
Giltay [28]	235	NR	X	NR	NR	NR	NR	NR	Skin abnormalities, burning eyes
De Jong [29]	42	X	X	X	X	X	NR	NR	Rashes, oral ulcers
Englert [30]	458	NR	NR	X	NR	NR	NR	X	Night sweats, breast pain, reflux, paraesthesiae, lethargy
Vasey [31]	50	X	X	X	NR	NR	NR	NR	Lymphadenopathy
Vermeulen [32]	319	X	X	X	NR	NR	NR	X	Headache, lymphadenopathy, sore throat
Bridges [33]	156	X	X	X	X	X	NR	X	Pulmonary symptoms, alopecia, ulcers, lymphadenopathy
Freundlich [34]	50	X	X	NR	X	NR	NR	NR	Raynaud, alopecia, lymphadenopathy, night sweats, sore throats
Solomon [35]	176	X	X	NR	X	NR	NR	X	Skin abnormalities, gland enlargement, dysphagia, carpel tunnel syndrome, alopecia
Peters [22]	100	X	X	X	NR	NR	NR	X	Gastrointestinal symptoms, breast pain, rashes
Mehmed [36]	240	X	X	NR	NR	X	X	X	Dysphagia, hair loss, depression, skin rash, headache
Wells [37]	52	X	X	X	NR	X	NR	X	Headaches, swollen glands, back pain, rashes

*X* present symptom, *NR* not reported

Our literature search yielded 390 citations. In total, 18 studies were included; 5 studies met eligibility criteria and were included. Thirteen studies were references in the previous included studies, met eligibility criteria and were included as well.

Maijers et al. [15] presented a cohort of 80 patients with silicone breast implants and unexplained symptoms such as fatigue, neurasthenia, myalgia, arthralgia, morning stiffness and night sweats in more than 60 % of women. In addition, women experienced cognitive problems, dermatological symptoms, gastrointestinal symptoms, alopecia, sleeping disorders and depression.

Cohen Tervaert et al. [3] presented a cohort of 32 patients with silicone breast implants. Patients presented with fatigue, arthralgia, myalgias, asthenia and/or fever. In addition, 50 % of patients had an immunodeficiency, whereas also 50 % had an autoimmune disease.

Contant et al. [23] prospectively evaluated a cohort of 57 women with silicone-related symptom complex that were seen in an breast cancer centre between March 1995 and March 1997, who presented with sicca symptoms (dry eyes and dry mouth), arthralgias/arthritis, Raynaud’s phenomenon, headache, dizziness, memory difficulties, palpitations, diarrhoea and transpiration.

In another study, Contant et al. evaluated the serology of 63 women with silicone-related symptom complex who presented with complaints as described in the above mentioned study of Contant. These patients were examined between September 1990 and May 1995 [24]. They found that 16 % of the patients were ANA positive, but there was no difference in symptoms between ANA-positive and ANA-negative patients.

Shoaib et al. presented 26 women with a systemic disease with central nervous system involvement after receiving silicone breast implants or silicone fluid injection, with symptoms suggesting a multiple sclerosis-like syndrome. Additional symptoms were myalgia, joint stiffness, arthralgia, sicca complex, headache, skin rash, joint swelling, Raynaud’s phenomena, fever, hair loss, allergies, sensitivity to sunlight and lymphadenopathy [25].

Cuellar et al. evaluated a cohort of 300 consecutive women seen in the Rheumatology department of the LSU Medical Center at New Orleans from January 1991 to November 1992 with silicone breast implants with musculoskeletal complaints: chronic fatigue, arthralgia, low-grade fever, sicca, memory loss, multiple sclerosis-like syndrome, night sweats, headaches, chronic diarrhoea, recurrent infections, dyspnoea and angio-oedema [26].

Gaubitz et al. [27] presented 90 consecutive symptomatic women with silicone breast implants who underwent an MRI to detect implant rupture. These patients presented with a scala of symptoms: fatigue, arthralgia, myalgia, night sweats, dry eyes, swollen joints, Raynaud, tingling, photosensitivity, headache, dyspnoea, depression and hoarseness. Clinical symptoms in patients with ruptured SBI did not differ from patients with an intact SBI.

Giltay et al. presented a study in which 235 patients with silicone breast implants and 210 healthy controls filled in a questionnaire reflecting complaints such as painful or swollen joints, burning eyes, oral ulcers, Raynaud’s phenomenon and/or skin abnormalities. Patients with silicone breast implant significantly reported more complaints after surgery than before surgery. Especially, more painful joints, burning eyes and skin abnormalities were reported compared to the control group [28].

De Jong et al. present a cohort of 42 symptomatic patients with silicone breast implants who were evaluated for antipolymer antibodies. These patients presented with symptoms as fatigue, arthralgia, morning stiffness, myalgia, sleep disturbance, rashes, dry eyes/mouth, oral ulcers, muscle weakness and fevers [29]. SBI exposure did not result in induction of polymer binding antibodies.

Englert et al. evaluated 458 female patients with silicone breast implants compared to a control group of 687 women who underwent plastic surgery but did not receive silicone breast implants. They found that patients with silicone breast implants more commonly had complaints such as night sweats, lethargy, breast pain, impaired mentation, reflux, paraesthesiae, hand muscle weakness and myalgia [30].

Vasey et al. presented the clinical findings in a cohort of 50 symptomatic patients with silicone breast implants. The most common clinical findings included chronic fatigue, muscle pain, joint pain, joint swelling and lymphadenopathy [31].

Vermeulen et al. studied the presence of symptoms of pain and fatigue in a cohort of 319 women with silicone breast implants. The four most frequent complaints in the women with implants were multi-joint pain, muscle pain, debilitating chronic fatigue and postexertional malaise. Fewer women had un-refreshing sleep, impaired cognition and headache, whereas one-third or fewer of the women with implants complained of painful lymph nodes and sore throat [32].

Bridges et al. clinically and immunologically evaluated 156 women with silicone breast implants and symptoms of a rheumatic disease. These patients presented with fatigue, myalgia, arthralgia, sicca symptoms, mental confusion, pulmonary symptoms, alopecia, recurrent fever, lymphadenopathy and mucosal ulcerations [33].

Freundlich et al. described typical sicca complaints in combination with a pattern of rheumatic symptoms (fatigue, generalized stiffness, poor sleep and arthralgias) in 50 female patients with silicone gel breast implants. They concluded that their patients did not fit into one single autoimmune, rheumatologic or neurological disease. Other problems in these patients included Raynaud’s phenomenon, alopecia, lymphadenopathy, night sweats and frequent sore throats [34].

Solomon et al. presented a clinical and laboratory profile of 176 symptomatic women with silicone breast implants. The most frequent symptoms seen in the women were chronic fatigue, cognitive dysfunction, arthralgia, dry mouth, dry eye, alopecia, dysphagia, telangiectasias, erythema of the chest wall, carpal tunnel syndrome, petechiae, lacrimal gland enlargement, thyroid tenderness, thyroid enlargement and parotid enlargement [35].

Peters et al. reported 100 female patients who underwent explantation of their breast implants. Patients had systemic symptoms such as arthralgia, myalgia, fatigue, gastrointestinal symptoms, rashes, memory loss, sleep disturbances and breast pain [22].

Mehmed et al. presented 240 patients undergoing explantation of their silicone breast implants. 196 patients underwent explantation with complaints such as chronic fatigue, memory loss, arthralgias, dysphagia, depression, altered sleep patterns, hair loss, skin rashes, headaches, flu-like symptoms and atypical multiple sclerosis [36].

Finally, Wells et al. presented 52 women requesting removal of their silicone breast implants because they suspected a relation with complaints such as arthralgia, fatigue, myalgia, headaches, fevers, swollen glands, back pain, rashes, memory loss and swollen joints [37].

## Summary of the reviewed literature

A summary of the clinical manifestations identified in the cohorts is given in Table 7. The clinical manifestations of ASIA major criterion 2 are used as guideline (see Table 1); other symptoms are summarized under the heading ‘other’.

After comparing the Maastricht cohort and the Baylor College cohort with the other identified studies, we concluded that a great similarity in clinical manifestations exists in all studies. Especially, the 7 typical clinical manifestations of ASIA (major criterion 2) are present throughout all identified cohorts. However, it should be noted that several other symptoms are frequently present, such as Raynaud’s phenomenon, headache, alopecia or hair loss, skin abnormalities, gastrointestinal symptoms (irritable bowel syndrome), night sweats and lymphadenopathy. These clinical findings in patients with silicone breast implants resemble the clinical picture of fibromyalgia [38–40]. It has been postulated that in fibromyalgia, nociceptive signals (often psychological traumas) cause the development of symptoms via disturbed pain processing [41]. We propose that in patients with ASIA due to SIIS, the breast implant might be the nociceptive stimulus. The nociceptive stimulus (silicone) in combination with extensive worrying about the safety of the breast implant causes a disturbed pain signalling pathway and excessive stimulation of neurotransmitters in the central nervous system and subsequently the systemic complaints [41]. A major difference between idiopathic fibromyalgia and silicone-induced fibromyalgia, however, is the co-occurrence of immune deficiency [3] and/or autoimmunity [3, 15, 42] during follow-up in patients with ASIA due to SIIS.

## Conclusion

Are silicone breast implants safe? After half a century of worldwide usage, this is still a matter of debate. In 1992, the FDA restricted the use of silicone breast implant in the USA due to reports of fibromyalgia-like health complaints, systemic symptoms and autoimmunity [43]. In 2000, Janowsky et al. [12] performed a meta-analysis and concluded that silicone breast implants could be considered safe. However, in this meta-analysis a study of 10.830 patients was excluded, due to the fact that the complaints of these patients were ‘self-reported’ [44]. If this study was not excluded, the relative risk for developing connective tissue disease would have increased from insignificant to significant with a value of 1.3 [44]. Furthermore, in the meta-analysis by Janowsky et al. [12] only the development of well-defined connective tissue disease was addressed, and not the development of ASIA, adjuvant breast disease or other less well-defined conditions. This should imply that despite changes in the principal constituents of the silicone implants during the past fifty years, silicone remained an adjuvant that may ‘bleed’ and subsequently may be a chronic stimulus to the immune system resulting in similar clinical manifestations as observed in the Maastricht cohort, the Baylor College cohort and 18 other large cohorts of patients.

In conclusion, we report that there is a group of patients who develop complaints related to silicone breast implants. In the past thirty years, the character of silicone-related complaints has been similar. Whether silicone breast implants are safe, or if they are only safe in a subgroup of female patients, is however, after more than these thirty years, still not clear. This should be studied in future epidemiological and experimental studies. This research should be conducted, because the current evidence that silicone breast implants are safe is at present limited.

## Abbreviations

ASIA: Autoimmune/inflammatory syndrome induced by adjuvants
SIIS: Silicone implant incompatibility syndrome
CTD: Connective tissue disease
ANA: Antinuclear antibodies
RF: Rheumatoid factor

## References

[1] The American Society for Aesthetic Plastic Surgery. National Plastic Surgery Statistics Editor. California: The American Society for Aesthetic Plastic Surgery. 2009.

[2] Miyoshi K, Miyamura T, Kobayashi Y, Itakura T, Nishijo K (1964). Hyper-gammaglobulinemia by prolonged adjuvanticity in man: disorders developed after augmentation mammaplasty. Jpn Med J.

[3] Cohen Tervaert JW, Kappel RM (2013). Silicone implant incompatibility syndrome (SIIS): a frequent cause of ASIA (Shoenfeld’s syndrome). Immunol Res.

[4] Lidar M, Agmon-Levin N, Langevitz P, Shoenfeld Y (2012). Silicone and scleroderma revisited. Lupus.

[5] Levy Y, Rotman-Pikielny P, Ehrenfeld M, Shoenfeld Y (2009). Silicone breast implantation-induced scleroderma: description of four patients and a critical review of the literature. Lupus.

[6] Bar-Meir E, Eherenfeld M, Shoenfeld Y (2003). Silicone gel breast implants and connective tissue disease: a comprehensive review. Autoimmunity.

[7] Zandman-Goddard G, Ehrenfeld M, Shoenfeld Y (1993). Silicone implants for breast augmentation and autoimmune diseases. Harefuah.

[8] Ehrenfeld M, Shoenfeld Y (1998). Breast silicone implant and autoimmunity: coincidence or cause and effect relationship?. Harefuah.

[9] Jewell M, Spear SL, Largent J, Oefelein MG, Adams WP (2011). Anaplastic large T-cell lymphoma and breast implants: a review of the literature. Plast Reconstr Surg.

[10] Bizjak M, Selmi C, Praprotnik S, Bruck O, Perricone C, Ehrenfeld M, Shoenfeld Y (2015). Silicone implants and lymphoma: the role of inflammation. J Autoimmun.

[11] Kadin ME, Deva A, Xu H, Morgan J, Khare P, MacLeod RA, van Natta BW, Adams WP Jr, Brody GS, Epstein AL. Biomarkers provide clues to early events in the pathogenesis of breast implant-associated anaplastic large cell lymphoma. Aesthet Surg J. 2016 10.1093/asj/sjw023 26979456

[12] Janowsky EC, Kupper LL, Hulka BS (2000). Meta-analyses of the relation between silicone breast implants and the risk of connective-tissue diseases. N Engl J Med.

[13] SCENIHR (Scientific Committee on Emerging and Newly Identified Health Risks). Preliminary opinion on the safety of Poly Implant Prothèse (PIP) Silicone Breast Implants (2013 update). September 2013. http://ec.europa.eu/health/scientific_committees

[14] Shoenfeld Y, Agmon-Levin N (2011). ‘ASIA’-autoimmune/inflammatory syndrome induced by adjuvants. J Autoimmun.

[15] Maijers MC, de Blok CJ, Niessen FB, van der Veldt AA, Ritt MJ, Winters HA (2013). Women with silicone breast implants and unexplained systemic symptoms: a descriptive cohort study. Neth J Med.

[16] Shoaib BO, Patten BM, Calkins DS (1994). Adjuvant breast disease: an evaluation of 100 symptomatic women with breast implants or silicone fluid injections. Keio J Med.

[17] Tervaert JW, Van Paassen P, Damoiseaux J (2007). Type II cryoglobulinemia is not associated with hepatitis C infection: the Dutch experience. Ann N Y Acad Sci.

[18] Damoiseaux JG, Tervaert JW (2006). From ANA to ENA: how to proceed?. Autoimmun Rev.

[19] Avery TY, van de Cruys M, Austen J, Stals F, Damoiseaux JG (2014). Anti-nuclear antibodies in daily clinical practice: prevalence in primary, secondary, and tertiary care. J Immunol Res.

[20] De Steenwinkel FD, Hokken-Koelega AC, de Ridder MA, Hazes JM, Dolhain RJ (2014). Rheumatoid arthritis during pregnancy and postnatal catch-up growth in the offspring. Arthritis Rheumatol.

[21] Bridges AJ, Conley C, Wang G, Burns DE, Vasey FB (1993). A clinical and immunologic evaluation of women with silicone breast implants and symptoms of rheumatic disease. Ann Intern Med.

[22] Peters W, Smith D, Fornasier V, Lugowski S, Ibanez D (1997). An outcome analysis of 100 women after explantation of silicone gel breast implants. Ann Plast Surg.

[23] Contant CM, Swaak AJ, Obdeijn AI, van der Holt B, Tjong Joe Wai R, van Geel AN (2002). A prospective study on silicone breast implants and the silicone-related symptom complex. Clin Rheumatol.

[24] Contant CM, Swaak AJ, Wiggers T, Wai RT, van Geel AN (2000). First evaluation study of the Dutch Working Party on silicone breast implants (SBI) and the silicone-related symptom complex (SRSC). Clin Rheumatol.

[25] Shoaib BO, Patten BM (1996). Human adjuvant disease: presentation as a multiple sclerosis-like syndrome. South Med J.

[26] Cuellar ML, Gluck O, Molina JF, Gutierrez S, Garcia C, Espinoza R (1995). Silicone breast implant–associated musculoskeletal manifestations. Clin Rheumatol.

[27] Gaubitz M, Jackisch C, Domschke W, Heindel W, Pfleiderer B (2002). Silicone breast implants: correlation between implant ruptures, magnetic resonance spectroscopically estimated silicone presence in the liver, antibody status and clinical symptoms. Rheumatol (Oxford)..

[28] Giltay EJ, Bernelot Moens HJ, Riley AH, Tan RG (1994). Silicone breast prostheses and rheumatic symptoms: a retrospective follow up study. Ann Rheum Dis.

[29] De Jong WH, Goldhoorn CA, Kallewaard M, Geertsma RE, Van Loveren H, Bijlsma JW, Schouten JS (2002). Study to determine the presence of antipolymer antibodies in a group of Dutch women with a silicone breast implant. Clin Exp Rheumatol.

[30] Englert H, Joyner E, Thompson M, Garcia H, Chambers P, Horner D, Hunt C, Makaroff J, O’Connor H, Russell N, March L (2004). Augmentation mammoplasty and “silicone-osis”. Intern Med J.

[31] Vasey FB, Havice DL, Bocanegra TS, Seleznick MJ, Bridgeford PH, Martinez-Osuna P, Espinoza LR (1994). Clinical findings in symptomatic women with silicone breast implants. Semin Arthritis Rheum.

[32] Vermeulen RC, Scholte HR (2003). Rupture of silicone gel breast implants and symptoms of pain and fatigue. J Rheumatol.

[33] Bridges AJ, Conley C, Wang G, Burns DE, Vasey FB (1993). A clinical and immunologic evaluation of women with silicone breast implants and symptoms of rheumatic disease. Ann Intern Med.

[34] Freundlich B, Altman C, Snadorfi N, Greenberg M, Tomaszewski J (1994). A profile of symptomatic patients with silicone breast implants: a Sjögrens-like syndrome. Semin Arthritis Rheum..

[35] Solomon G (1994). A clinical and laboratory profile of symptomatic women with silicone breast implants. Semin Arthritis Rheum.

[36] Melmed EP (1998). A review of explantation in 240 symptomatic women: a description of explantation and capsulectomy with reconstruction using a periareolar technique. Plast Reconstr Surg.

[37] Wells KE, Roberts C, Daniels SM, Kearney RE, Cox CE (1995). Psychological and rheumatic symptoms of women requesting silicone breast implant removal. Ann Plast Surg.

[38] Bennet RM, Jones J, Turk DC, Russel IJ, Matallana L (2007). An internet survey of 2,596 people with fibromyalgia. BMC Musculoskelet Disord.

[39] Rodriguez-Rodriguez L, Ramon Lamas J, Abasolo L (2015). The rs3771863 single nucleotide polymorphism of th TACR1gene is associated to a lower risk of sicca syndrome in fibromyalgia patients. Clin Exp Rheumatol Suppl.

[40] Wolfe F (1999). Silicone related symptoms” are common in patients with fibromyalgia: no evidence for a new disease. J Rheumatol.

[41] Clauw DJ, Arnold LM, McCarberg BH (2011). The science of fibromyalgia. Mayo Clin Proc.

[42] Agmon-Levin N, Shoenfeld Y (2008). Chronic fatigue syndrome with autoantibodies—the result of an augmented adjuvant effect of hepatitis-B vaccine and silicone implant. Autoimmun Rev.

[43] Kessler DA (1992). The basis of the FDA’s decision on breast implants. N Engl J Med.

[44] Soriano A, Butnaru D, Shoenfeld Y (2014). Long-term inflammatory conditions following silicone exposure: the expanding spectrum of the autoimmune/inflammatory syndrome induced by adjuvants (ASIA). Clin Exp Rheumatol.

